# ICTV Virus Taxonomy Profile: *Tulasviridae* 2023

**DOI:** 10.1099/jgv.0.001933

**Published:** 2023-12-20

**Authors:** Jens H. Kuhn, Scott Adkins, Katherine Brown, Juan Carlos de la Torre, Michele Digiaro, Holly R. Hughes, Sandra Junglen, Amy J. Lambert, Piet Maes, Marco Marklewitz, Gustavo Palacios, Takahide Sasaya, Yong-Zhen Zhang, Massimo Turina

**Affiliations:** ^1^​ Integrated Research Facility at Fort Detrick, National Institute of Allergy and Infectious Diseases, National Institutes of Health, Fort Detrick, Frederick MD 21702, USA; ^2^​ United States Department of Agriculture, Agricultural Research Service, US Horticultural Research Laboratory, Fort Pierce, FL 34945, USA; ^3^​ Division of Virology, Department of Pathology, Addenbrookes Hospital, University of Cambridge, Cambridge CB2 0QN, UK; ^4^​ Department of Immunology and Microbiology IMM-6, The Scripps Research Institute, La Jolla CA 92037, USA; ^5^​ CIHEAM, Istituto Agronomico Mediterraneo di Bari, 70010 Valenzano, Italy; ^6^​ Centers for Disease Control and Prevention, Fort Collins CO 80521, USA; ^7^​ Institute of Virology, Charité-Universitätsmedizin Berlin, Corporate Member of Freie Universität Berlin, Humboldt-Universität zu Berlin, and Berlin Institute of Health, Berlin 10117, Germany; ^8^​ KU Leuven, Rega Institute, Zoonotic Infectious Diseases Unit, 3000 Leuven, Belgium; ^9^​ FIND, 1202 Geneva, Switzerland; ^10^​ Department of Microbiology, Icahn School of Medicine at Mount Sinai, New York, NY 10029, USA; ^11^​ Institute for Plant Protection, National Agriculture and Food Research Organization, Tsukuba, Ibaraki 305-8517, Japan; ^12^​ School of Life Sciences and Human Phenome Institute, Fudan University, Shanghai 201052, PR China; ^13^​ Institute for Sustainable Plant Protection, National Research Council of Italy (IPSP-CNR), 10135 Torino, Italy

**Keywords:** ICTV Report, taxonomy, *Tulasviridae*, orthotulasvirus, Tulasnella bunyavirales-like virus 1

## Abstract

*Tulasviridae* is a family of ambisense RNA viruses with genomes of about 12.2 kb that have been found in fungi. The tulasvirid genome is nonsegmented and contains three open reading frames (ORFs) that encode a nucleoprotein (NP), a large (L) protein containing an RNA-directed RNA polymerase (RdRP) domain, and a protein of unknown function (X). This is a summary of the International Committee on Taxonomy of Viruses (ICTV) Report on the family *Tulasviridae*, which is available at ictv.global/report/tulasviridae.

## Virion

Unknown.

## Genome

**Fig. 1.**
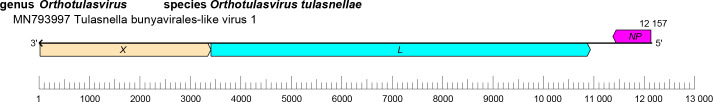
Genome organisation of Tulasnella bunyavirales-like virus 1. ORFs are coloured according to the predicted protein function (*L*, large protein gene; *NP*, nucleoprotein; *X*, gene encoding a protein of unknown function).

The tulasvirid genome comprises nonsegmented, linear, ambisense RNA with a total length of about 12.2 kb and three ORFs that encode an NP, an X, and an L protein containing an RdRP domain [1, 2] (Table 1, Fig. 1).

**Table 1.** Characteristics of members of the family *Tulasviridae*

Example	Tulasnella bunyavirales-like virus 1 (MN793997), species *Orthotulasvirus tulasnellae*, genus *Orthotulasvirus*
Virion	Unknown
Genome	About 12.2 kb of nonsegmented ambisense RNA
Replication	Unknown
Translation	Unknown
Host range	Agaricomycete fungi
Taxonomy	Realm *Riboviria*, kingdom *Orthornavirae*, phylum *Negarnaviricota*, class *Ellioviricetes*, order *Bunyavirales*; the family includes the genus *Orthotulasvirus* and the species *Orthotulasvirus tulasnellae*

## Replication

Unknown.

## Taxonomy

**Fig. 2.**
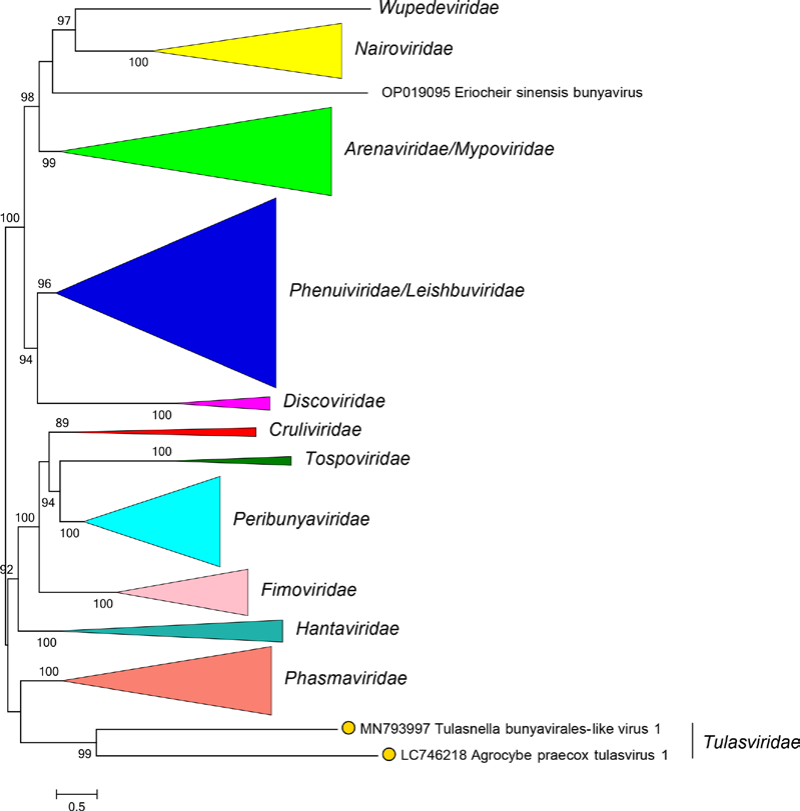
Phylogenetic relationships of Tulasnella bunyavirales-like virus 1. Family branches are collapsed. Numbers at nodes indicate bootstrap support >70 %. Details of viruses and methods are available in the full ICTV Report on the family *Tulasviridae*.

Current taxonomy: ictv.global/taxonomy. Tulasvirids are most closely related to crulivirids, fimovirids, hantavirids, peribunyavirids, phasmavirids, and tospovirids [3] (Fig. 2). The family includes the genus *Orthotulasvirus* for viruses that infect fungi. In contrast to most closely related viruses [4, 5], tulasvirids have nonsegmented, negative-sense single-stranded RNA genomes but encode proteins with high sequence identity to proteins of other bunyavirals and have five conserved motifs (A–E) in their RdRP domain.

## Resources

Full ICTV Report on the family *Tulasviridae*: ictv.global/report/tulasviridae.
